# Gene-edited *Mtsoc1* triple mutant Medicago plants do not flower

**DOI:** 10.3389/fpls.2024.1357924

**Published:** 2024-02-26

**Authors:** Axel Poulet, Min Zhao, Yongyan Peng, FangFei Tham, Mauren Jaudal, Lulu Zhang, Josien C. van Wolfswinkel, Joanna Putterill

**Affiliations:** ^1^ Department of Molecular, Cellular and Developmental Biology, Faculty of Arts and Sciences, Yale University, New Haven, CT, United States; ^2^ Flowering Lab, School of Biological Sciences, University of Auckland, Auckland, New Zealand; ^3^ Mt Albert Research Centre, The New Zealand Institute for Plant and Food Research Limited, Auckland, New Zealand

**Keywords:** MtSOC1a, MtSOC1b, MtSOC1c, legume, Medicago, flowering time, gene editing, CRISPR-Cas9

## Abstract

Optimized flowering time is an important trait that ensures successful plant adaptation and crop productivity. *SOC1-like* genes encode MADS transcription factors, which are known to play important roles in flowering control in many plants. This includes the best-characterized eudicot model *Arabidopsis thaliana* (Arabidopsis), where *SOC1* promotes flowering and functions as a floral integrator gene integrating signals from different flowering-time regulatory pathways. *Medicago truncatula* (Medicago) is a temperate reference legume with strong genomic and genetic resources used to study flowering pathways in legumes. Interestingly, despite responding to similar floral-inductive cues of extended cold (vernalization) followed by warm long days (VLD), such as in winter annual Arabidopsis, Medicago lacks FLC and CO which are key regulators of flowering in Arabidopsis. Unlike Arabidopsis with one *SOC1* gene, multiple gene duplication events have given rise to three *MtSOC1* paralogs within the *Medicago* genus in legumes: one Fabaceae group A *SOC1* gene, *MtSOC1a*, and two tandemly repeated Fabaceae group B *SOC1* genes, *MtSOC1b* and *MtSOC1c*. Previously, we showed that *MtSOC1a* has unique functions in floral promotion in Medicago. The *Mtsoc1a Tnt1* retroelement insertion single mutant showed moderately delayed flowering in long- and short-day photoperiods, with and without prior vernalization, compared to the wild-type. In contrast, *Mtsoc1b Tnt1* single mutants did not have altered flowering time or flower development, indicating that it was redundant in an otherwise wild-type background. Here, we describe the generation of *Mtsoc1a Mtsoc1b Mtsoc1c* triple mutant lines using CRISPR-Cas9 gene editing. We studied two independent triple mutant lines that segregated plants that did not flower and were bushy under floral inductive VLD. Genotyping indicated that these non-flowering plants were homozygous for the predicted strong mutant alleles of the three *MtSOC1* genes. Gene expression analyses using RNA-seq and RT-qPCR indicated that these plants remained vegetative. Overall, the non-flowering triple mutants were dramatically different from the single *Mtsoc1a* mutant and the Arabidopsis *soc1* mutant; implicating multiple *MtSOC1* genes in critical overlapping roles in the transition to flowering in Medicago.

## Introduction

Optimal timing of flowering is a major adaptive trait in plants and a key determinant of productivity in crops, including legumes (Graham and Vance, 2003; Jung and Muller, 2009; Tadege et al., 2015; Weller and Ortega, 2015; Foyer et al., 2016). *Medicago truncatula* (Medicago) is a temperate legume with powerful genetic and genomic resources for investigating the molecular pathways underlying flowering (Benlloch et al., 2006; Tadege et al., 2009; Young et al., 2011; Putterill et al., 2013; Meng et al., 2017; Cañas and Beltrán, 2018; Weller and Macknight, 2018; Jaudal et al., 2020a). Interestingly, Medicago and *Arabidopsis thaliana* (Arabidopsis), share similarities and striking differences in their flowering time regulation. Multiple signalling pathways control Arabidopsis flowering (Kim et al., 2009; Fornara et al., 2010; Srikanth and Schmid, 2011; Andrés and Coupland, 2012). Both winter annual Arabidopsis and Medicago are induced to flower by extended winter cold (vernalization, V) followed by warm, long day (LD) photoperiods (VLD) (Clarkson and Russell, 1975). However, Medicago lacks the well-known regulators of these processes, the repressor FLOWERING LOCUS C (FLC), and the activator CONSTANS (CO) (Putterill et al., 2013; Wong et al., 2014; Weller and Macknight, 2018; Jaudal et al., 2020a).

Nevertheless, progress has been made in identifying Medicago flowering-time regulators using forward and reverse genetics. These include the LD activators *MtPHYTOCHROME A* (Jaudal et al., 2020b), *MtFE* (Thomson et al., 2021), and *MtPHYTOCHROMOBILIN SYNTHASE* (Perez-Santangelo et al., 2022), and repressors *MtCYCLING DOF FACTORS* genes (Zhang et al., 2019). The polycomb repressive complex 2 component MtVERNALISATION2 represses Medicago flowering until after vernalization (Jaudal et al., 2016). *MtINHIBITOR OF GROWTH 2* (*MtING2*) promoted flowering particularly in response to VLD (Jaudal et al., 2022). The floral integrator genes include the *FLOWERING LOCUS T (FT)-like* gene *MtFTa1* (Laurie et al., 2011; Yeoh et al., 2013; Jaudal et al., 2020a; Zhang et al., 2021b) and the *FLOWERING LOCUS D (FD)-like* gene *MtFDa.* MtFDa interacts with MtFTa1 and stimulates the transition to flowering, as has been observed for Arabidopsis FT and FD (Srikanth and Schmid, 2011; Collani et al., 2019; Cheng et al., 2021; Zhang et al., 2021b; Zhu et al., 2021). *Mtfta1 Mtfda* double mutants have a bushy phenotype and fail to transition to flowering, indicating complementary critical functions for these genes in flowering time (Laurie et al., 2011; Cheng et al., 2021). This is unlike Arabidopsis, where the *ft-10 twin sister of ft (tsf-1) fd-3* triple mutants showed only delayed flowering (Romera-Branchat et al., 2020). Other likely players include multiple paralogs of genes encoding MADS transcription factors, such as the three *MtSUPPRESSOR OF OVEREXPRESSION OF CO1 (SOC1)* (*MtSOC1a*, *b*, *c*), three *MtFUL* genes (*MtFULa, b, c*), three *MtSHORT VEGETATIVE PHASE* (*MtSVP1, 2, c*), and additional *FT*/*TERMINAL FLOWER 1 (TFL1)* genes including *MtFTa2*, *MtFTb1*, *MtFTb2*, and *MtTFL1a, c* (Laurie et al., 2011; Jaudal et al., 2014, 2015; Sussmilch et al., 2017; Cheng et al., 2018; Fudge et al., 2018; Jaudal et al., 2018; Thomson et al., 2019; Jaudal et al., 2022).

*SOC1* functions as an important floral integrator gene in Arabidopsis, integrating signals from different flowering time pathways, including the LD photoperiod, gibberellin (GA), ambient temperature, and age pathways (Melzer et al., 2008; Gregis et al., 2009; Liu et al., 2009; Lee and Lee, 2010; Balanzà et al., 2014; Romera-Branchat et al., 2020). SOC1 binds to and controls the expression of numerous flowering regulators, including *SVP*, *APETALA* (*AP2)-like* repressor genes, and floral homeotic genes, and represses its own expression (Immink et al., 2012; Tao et al., 2012). *SOC1* homologues regulate flowering or phenotypes, such as dormancy, in other plants (Lee and Lee, 2010; Immink et al., 2012; Tao et al., 2012; Voogd et al., 2015; Jaudal et al., 2018). Medicago has three *MtSOC1* genes that encode proteins with ~66% amino acid identity with SOC1. These include the Fabaceae group A *SOC1* gene, *MtSOC1a*, on chromosome 7, and two Fabaceae group B *SOC1* genes, *MtSOC1b* and *MtSOC1c*, adjacent to each other on chromosome 8 (Fudge et al., 2018; Jaudal et al., 2018). The latter shares 93% amino acid identity and is ~66% identical to MtSOC1a. *MtSOC1b* and *MtSOC1c* duplications have only been observed in the genus Medicago, suggesting that it is relatively recent (Fudge et al., 2018; Jaudal et al., 2018). The three *MtSOC1* genes partially complemented the delayed flowering of the Arabidopsis *soc1* mutant (Fudge et al., 2018) and *MtSOC1a* overexpression strongly accelerated flowering in some wild-type (WT) Arabidopsis transgenic plants (Jaudal et al., 2018). In Medicago, the three *MtSOC1* genes showed elevated expression in the shoot apex in response to floral inductive signals of VLD (Fudge et al., 2018; Jaudal et al., 2018). In addition, their expression was reduced in the late-flowering *Mtfta1* mutant but elevated in transgenic plants overexpressing *MtFTa1*. This indicates that their expression relies partly on functional *MtFTa1*, as observed for the *SOC1*-mediated LD promotion of flowering in Arabidopsis (Fudge et al., 2018; Jaudal et al., 2018). Recently, a role for *MtFDa* in promoting *MtSOC1* gene expression was reported, with the three *MtSOC1* genes showing reduced expression in the *Mtfda* late-flowering mutant (Cheng et al., 2021, Zhang et al., 2021b).

In our previous study, we showed that *MtSOC1a* promotes flowering in Medicago, and that overexpression of *MtSOC1a* partially rescued the late flowering phenotype of the *Mtsoc1a Tnt1* insertion mutant (Jaudal et al., 2018). However, *Mtsoc1b Tnt1* insertion mutants had no flowering time phenotype, suggesting that *MtSOC1b* function may be redundant in regulating flowering in the WT (Fudge et al., 2018; Jaudal et al., 2018). *Mtsoc1c Tnt1* mutants have not been previously identified; however, overexpression of *MtSOC1c* in WT R108 accelerated Medicago flowering under LD conditions, indicating that it is likely involved in floral induction (Fudge et al., 2018; Jaudal et al., 2018; Yuan et al., 2023).

To analyze the combined functions of the three *MtSOC1* genes, we generated *Mtsoc1a Mtsoc1b Mtsoc1c* triple mutant lines using CRISPR/Cas9 gene editing. Strikingly, two independent triple mutant lines segregated plants that did not flower and were bushy, even after 5 months, compared with WT plants that flowered at ~3–4 weeks in VLD conditions. RNA-seq and RT-qPCR were carried out to analyze the molecular basis of these phenotypes and indicated that the non-flowering mutant plants remained vegetative, implicating a combined critical role for multiple *MtSOC1* genes in promoting the transition to flowering.

## Materials and methods

### Plant materials, growth conditions, and phenotyping

The wild-type (WT) Medicago plants R108_C3 (R108) (Trinh et al., 1998) and the *Tnt1* insertion *Mtsoc1a* (NF1705) single mutant in the R108 background have been previously reported (Jaudal et al., 2018). The gene-edited *Mtsoc1a Mtsoc1b Mtsoc1c* triple mutant lines, named *Mtsoc1-1* and *Mtsoc1-2*, were generated in this study in the WT background, as described below. For the typical growth of Medicago plants under floral-inductive vernalized long-day conditions (VLD), seeds were first scarified between two pieces of sandpaper (grade P600), sterilized in a chlorine solution (Millipore, USA) for 5 min–10 min, and germinated overnight at 15°C in the dark. For the vernalization treatment, germinated seeds were placed on moist filter paper and stored at 4°C in the dark for 3 weeks. Germinated seeds with or without vernalization were planted in seed-raising mix (Daltons Limited, NZ) and placed on rockwool mats subirrigated with hydroponics (Gibeaut et al., 1997, without Na_2_O_3_Si). Seedlings were transplanted after 11–14 days to 2 L pots of soil mix consisting of nine parts of potting mix (Daltons Limited, NZ), three parts of vermiculite (Pacific Growers Supplies Limited, NZ), one part of number 2 sand (Daltons Limited, NZ). Plants were grown in controlled rooms under long days (LD, 16 h light/8 h dark) at 22°C with fluorescent lights at ~160 μmol m^−2^s^−1^. Plants were watered with tap water and hydroponic medium.

Flowering time was scored in days after planting and the number of nodes on the primary axis at the time the first floral bud was observed on the plant. The length of the primary shoot axis (main stem) was measured from the monofoliate leaf node to the uppermost shoot apical bud and the number of nodes was counted. The longest secondary axis that branched off from the primary shoot axis was also identified, and its length and number of nodes were measured. The flowering time and shoot axis measurements are shown as box plots. Statistical significance was determined using the Wilcoxon test (*P*-value with Bonferroni correction: *P ≤0.05, **P ≤0.01, ***P ≤0.001, ****P ≤0.0001).

### General bioinformatics

Medicago gene sequences *MtSOC1a* (Medtr7g075870), *MtSOC1b* (Medtr8g033250), and *MtSOC1c* (Medtr8g033220) were obtained from the ‘Jemalong A17’ accession in the *M. truncatula* Genome Database (Mt4.0v2) http://blast.jcvi.org/Medicago-Blast/index.cgi) (Tang et al., 2014). The WT R108 genome assembly (v1.0, http://blast.jcvi.org/Medicago-Blast/index.cgi) (Moll et al., 2017) was used for the primer design and gene editing. Primers were designed using the Geneious software package (≥v8.0) (Kearse et al., 2012) using the Primer3 plugin (Untergasser et al., 2012).

### Generation of CRISPR-Cas9 gene-edited *Mtsoc1* triple mutant lines

Gene editing was used to generate the *Mtsoc1a Mtsoc1b Mtsoc1c* triple mutant lines. Two triple mutant lines were studied further and were named *Mtsoc1-1* and *Mtsoc1-2*. Seven single guide RNAs (guides) were designed using the Geneious Prime software (version 2019.1.1) (Kearse et al., 2012) to target the coding sequences of *MtSOC1a*, *MtSOC1b*, and *MtSOC1c* in Medicago WT A17 (**Table 1**; **Supplementary Table 1**). Two guides (guide 1 and guide 2) targeted exons 5 and 7 of *MtSOC1a*, which encode part of the K-domain and C-terminal domain of MtSOC1a, respectively. Two guides (guides 3 and 4) targeted *MtSOC1b* in exons 3 and 7, which encode a part of the K-domain and C-terminal domain of MtSOC1b, respectively. Four guides (guide 3, guide 5, guide 6, and guide 7) targeted exons 3, 4, 5, and 7 of *MtSOC1c*, respectively. These exons encode part of the K-domain (guides 3, 5, and 6) or the C-terminal domain (guide 7) of MtSOC1c. The guide sequences are listed in **Supplementary Table 1**. The guides were identified based on N(17)VVR(NGG)H target selection criteria and activity scoring by Doench et al. (2014), and checked for the absence of off-target sites in the Medicago R108 genome (v1.0). One construct consisting of a polycistronic pre-tRNA-sgRNA-scaffold cassette (Xie et al., 2015) placed downstream of the *Medicago* U6 promoter with all seven guides and HindIII restriction sites added on both ends was commercially synthesized by GenScript (USA). The construct was inserted into the pCBSG041 plasmid vector backbone with Cas9 driven by the constitutive CAMV 35S promoter, as previously described (Jaudal et al., 2022). The plasmid was transformed into *Agrobacterium tumefaciens* strain EHA105 to transform Medicago WT R108 leaf tissue. Independent T0 transformant plants were selected using phosphinothricin (PPT) and regenerated as described previously (Cosson et al., 2006; Jaudal et al., 2018). Young regenerant plants were also sprayed with the Basta herbicide (Bayer, Germany) to select transgenic plants. T0 mutant plants were self-crossed to produce segregating T1 and T2 progenies for analysis. *MtSOC1a*, *MtSOC1b*, and *MtSOC1c* were genotyped for gene editing by polymerase chain reaction (PCR) using the primers listed in **Supplementary Table 1**. The identity of the edits in *MtSOC1* genes was confirmed by DNA sequencing of the PCR products (Macrogen, South Korea).

**Table 1:** Summary of CRISPR-Cas9 gene edits in *Mtsoc1a*, *Mtsoc1b*, and *Mtsoc1c* alleles segregating in *Mtsoc1-1* and *Mtsoc1-2* triple mutant lines.

*Mtsoc1-1* triple mutant line							
Alleles	g1	g2	g3	g4	g5	g6	g7
	*SOC1a*, ex5, K	*SOC1a*, ex7, C	*SOC1b* & *c*, ex3, K	*SOC1b*, ex7, C	*SOC1*c, ex4, K	*SOC1c*, ex5, K	*SOC1c*, ex7, C
***Mtsoc1a-1***	Δ70 T171-A240	Did not amplify	*	*	*	*	*
***Mtsoc1a-2***	WT	+A A1135_C1136	*	*	*	*	*
***Mtsoc1b-1***	*	*	Δ78 C434-T511, +37 A433_T512	WT	*	*	*
***Mtsoc1b-2***	*	*	WT	WT	*	*	*
***Mtsoc1c-1***	*	*	WT	*	WT	Δ36 G511-A546	Δ38 T1582-T1619
***Mtsoc1c-2***	Unknown structural rearrangement; external c-F1 primer in intron 2 was unable to amplify the gDNA.						
*Mtsoc1-2* triple mutant line							
Alleles	g1	g2	g3	g4	g5	g6	g7
***Mtsoc1a-3***	WT	+G A1134_A1135	*	*	*	*	*
***Mtsoc1a-4***	Δ4 G219-A222	Δ2 A1134-A1135	*	*	*	*	*
***Mtsoc1b-3***	*	*	WT	Δ6 T1668-A1673	*	*	*
***Mtsoc1b-4***	*	*	Δ2 C466-A467/Inv1202 A468-C1669/Δ2 A1670-A1671		*	*	*
***Mtsoc1c-3***	*	*	WT	*	Δ199 T323-G521		Δ4 G1606-C1609
***Mtsoc1c-4***	*	*	Δ3 T163-C165	*	Δ3 A322-G324	Δ6 A518-A523	ΔA1607

The seven guides are indicated as g1–g7. The MtSOC1 gene(s) they target, location by exon (ex) and corresponding encoded MADs transcription factor domains (Keratin-like, K; C-terminal, C) are indicated. The first nucleotides of the F1 primers (**Supplementary Table 1**) are treated as +1. Δ, Deletion; +, Insertion; −, From left to right nucleotide, inclusively; _, Between left nucleotide and right nucleotide; *, Not applicable.

### Plant tissue harvesting and RNA extraction

For RNA-seq of a single *Mtsoc1a* mutant, apex samples were harvested from 15-day old *Mtsoc1a* and WT plants. The upper portion of three primary stems and their shoot apices, from three plants were pooled to make up one biological replicate, with three biological replicates harvested for each genotype. For real time reverse transcription quantitative PCR (RT-qPCR) and RNA-seq on the *Mtsoc1a Mtsoc1b Mtsoc1c* triple mutant line *Mtsoc1-2*, shoot apices from non-flowering *Mtsoc1-2* plants and WT plants were harvested. Three primary shoot apices from three *Mtsoc1-2* plants were harvested at 83 days (after phenotyping) and pooled to make up one biological replicate, with three biological replicates harvested. WT shoot apex samples for RT-qPCR comparisons with *Mtsoc1-2* plants were harvested on day 14. Three biological replicates were harvested, each consisting of three primary apices. Shoot apices (apex) (15 mg–100 mg), were harvested from plants grown under VLD conditions at zeitgeber time 4 (4 h after dawn). Tissues were snap-frozen with metal beads and homogenized using a Geno/Grinder (New Jersey, USA) into powder in liquid nitrogen. Total RNA was extracted using an RNeasy Plant Mini Kit (Qiagen, Germany) following the manufacturer’s protocol. The quantity and quality of the RNA were checked using a Bioanalyzer 2100 (Agilent Technologies, USA).

### Gene expression analysis by RT-qPCR

The WT and *Mtsoc1-2* triple mutant apex RNA samples were treated with DNase (TURBO DNA-free Kit; Invitrogen) before being subjected to cDNA synthesis and RT-qPCR, as previously described (Laurie et al., 2011; Zhang et al., 2019). Primer sequences used for RT-qPCR are listed in **Supplementary Table 1**. Relative gene expression was calculated based on the comparative Ct method (Livak and Schmittgen, 2001), with modifications (Bookout and Mangelsdorf, 2003), using the formula 2^−ΔCT^, where ΔCT was obtained by normalizing the gene of interest to the reference gene, *PROTEIN PHOSPHATASE 2A* (PP2A, Medtr6g084690). Statistical significance was calculated using the t-test, assuming unequal variance (p ≤0.05).

### RNA-seq and bioinformatic analysis

Shoot apex RNA samples of the *Mtsoc1a* single mutant and WT were sent to Novogene (Hong Kong) and six strand-specific mRNA libraries with polyA enrichment were prepared and subjected to Illumina NovaSeq 6000, 150 bp paired-end sequencing. Similarly, three strand-specific mRNA libraries with polyA enrichment were prepared from the shoot apex RNA of the non-flowering plants of the *Mtsoc1-2* mutant line. The FASTQ file read quality was evaluated, and the Fastp version (0.21) was used for trimming (Chen et al., 2018). When quality was below the PHRED score of 20, reads were trimmed from the 3’end, and reads <36bp in length were excluded. The remaining reads were mapped against the Mt4.0v2 transcriptome (Young et al., 2011; Tang et al., 2014) using Salmon (v0.8.2) (Patro et al., 2017). The resulting count tables were imported into R (R Core Team, 2018) using the tximport package (v1.12.0) (Soneson et al., 2015). DESeq2 (v1.24.0) (Love et al., 2014) was used for normalization and differential expression analyses. Differentially expressed transcripts were filtered using a cut-off adjusted p-value ≤0.05 and log2 fold-change ≥1 or ≤−1. To identify candidate direct target genes of MtSOC1s, we used Blastx (Altschul et al., 1990) against Arabidopsis SOC1 bound and regulated genes (Immink et al., 2012). All the genes with an e-value <e−100 were selected, and we removed the genes that were not expressed or weakly expressed, which gave a list of 253 transcripts (**Supplementary Table 4**). Raw RNA-seq data were available from GEO (GSE247931).

### Gene ontology enrichment analysis

ShinyGO v0.77 (Ge et al., 2020) was used for gene ontology enrichment analysis of the differentially expressed genes that are significantly over-represented in biological processes, cellular components, and molecular functions. *Medicago* genes (MedtrA17_4.0) and a false discovery rate p-value cut-off 0.05 was used.

## Results

### CRISPR-Cas9 gene editing to generate *Mtsoc1a Mtsoc1b Mtsoc1c* triple mutants

To investigate the combined function of the three *MtSOC1* genes *MtSOC1a*, *MtSOC1b*, and *MtSOC1c* in Medicago development and flowering, we created a CRISPR-Cas9 gene editing vector with seven guides to generate triple mutant plants. These targeted the exons that encode a part of the MADS transcription factor conserved K domain or the variable C domain (**Supplementary Table 1**, **Table 1**) (Lai et al., 2021). Of the seven guides, two (guides 1 and 2) specifically targeted *MtSOC1a*, one (guide 3) targeted both *MtSOC1b* and *MtSOC1c*, one (guide 4) targeted *MtSOC1b* and three (guides 5–7) targeted *MtSOC1c*, (**Supplementary Table 1**, **Table 1**). WT R108 Medicago leaf explants were transformed using *Agrobacterium*-mediated gene transfer, and T0 transgenic plants were regenerated via somatic embryogenesis. The transformants were genotyped to identify plants with *Mtsoc1a*, *Mtsoc1b*, and *Mtsoc1c* mutations. This was followed by further analyses to segregate T1 and T2 progeny generations. Two independent *Mtsoc1a Mtsoc1b Mtsoc1c* triple mutant lines were analyzed. These lines were named *Mtsoc1-1* and *Mtsoc1-2*, respectively (**Table 1**).

### The *Mtsoc1* triple mutant lines segregate plants that are non-flowering

**Figure 1:**
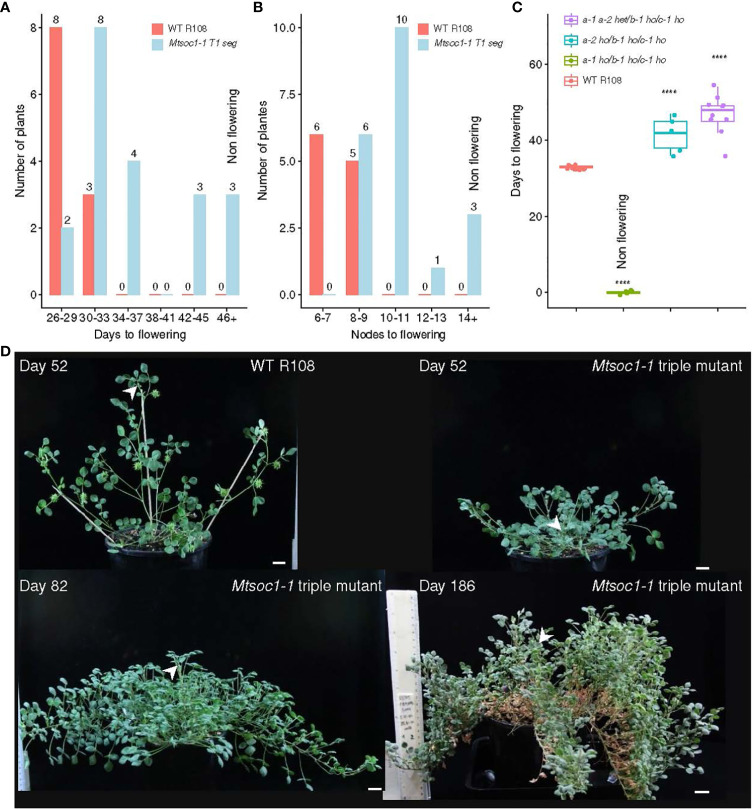
The *Mtsoc1a Mtsoc1b Mtsoc1c* triple mutant line *Mtsoc1-1* segregates plants that do not flower. **(A, B)** Distribution graphs in days **(A)** and node numbers on the primary axis **(B)** of the first flower of WT R108 (WT) and *Mtsoc1-1* T1 segregating line (R545) under VLD. The sample sizes are shown above each bar **(C)** Box plots showing the number of days to first flower of the WT and *Mtsoc1-1* T2 segregating lines (R760 and R761) under VLD. The homozygous (ho) and heterozygous (het) genotypes are shown. Statistical significance was determined using the Wilcoxon test (*P*-value with Bonferroni correction: ****P ≤0.0001). **(D)** Photographs of WT and non-flowering *Mtsoc1-1* triple mutants (R656-6, right top and left bottom, and R545-2, right bottom) under VLD. The white stealth arrows indicate the apex of the primary axis. Scale is 2 cm.

**Figure 2:**
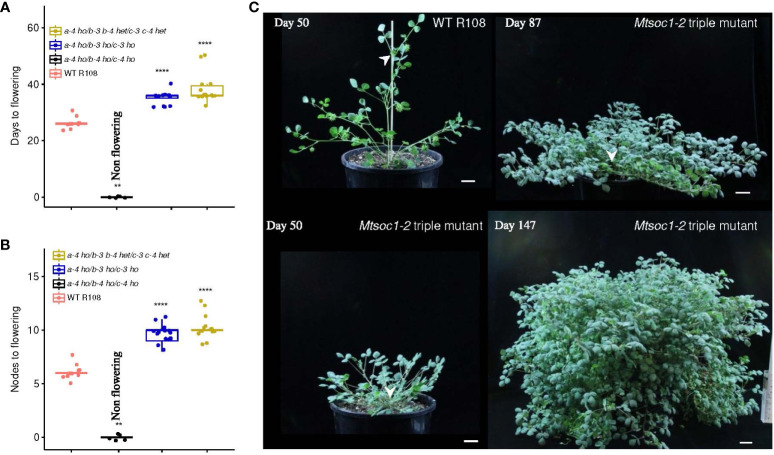
The triple mutant line *Mtsoc1-2* segregates non-flowering plants. **(A, B)** Box plots showing the number of days **(A)** and node number on the primary axis **(B)** to the first flower of the WT and *Mtsoc1-2* T2 segregating line (R647) under VLD. Homozygous (ho) and heterozygous (het) genotypes are shown. Statistical significance was determined using the Wilcoxon test (*P*-value with Bonferroni correction: ****P ≤0.0001). **(C)** Photographs of the WT and non-flowering *Mtsoc1-2* triple mutant (R647-26) taken on days 50, 87, and 147 under VLD. White stealth arrows indicate the apices of the primary axis. Scale is 2 cm.

The flowering time phenotypes of plants from the two triple mutant lines *Mtsoc1-1* and *Mtsoc1-2* were compared with those of WT plants under VLD. These lines segregated non-flowering plants (**Figures 1A–D**, **2A–C**; **Supplementary Figures 1A, B**; **Supplementary Table 2**). Strikingly, the latter plants did not flower even when grown for 5 months or more, compared to WT plants that flowered much earlier at ~1 month of age. *Mtsoc1-1* T1 plants segregated three plants with a non-flowering phenotype (**Figures 1A, B, D**). The remaining 17 siblings generally showed delayed flowering, ranging from 28 to 42 days, with increased node numbers on the primary axis (**Figures 1A, B**), similar to the *Mtsoc1a Tnt1* single mutant (Jaudal et al., 2018). One plant with the non-flowering phenotype was again observed in a separate sowing of *Mtsoc1-1* T1 plants, and five non-flowering plants were observed out of the 20 plants in the progeny T2 line tested (**Figure 1C**; **Supplementary Figures 1A, B**). Genotyping by PCR and DNA sequencing indicated 100% co-segregation between the non-flowering phenotype and homozygosity of the predicted strong deleterious mutant alleles *Mtsoc1a-1*, *Mtsoc1b-1*, and *Mtsoc1c-1*, with large deletions at one or more guide target sites in each gene (**Table 1**; **Supplementary Figure 1**; **Supplementary Table 2**). Genotyping and segregation analyses also indicated that the specific *Mtsoc1b* and *Mtsoc1c* alleles were inherited together, as predicted for genes adjacent to each other on the same chromosome. Thus, the *Mtsoc1b-1* and *Mtsoc1c-1* alleles were tightly linked, as were *Mtsoc1b-2* (wild-type allele) and *Mtsoc1c-2* (**Table 1**; **Supplementary Table 2**).

In the second triple mutant line, *Mtsoc1-2*, five non-flowering T2 plants were observed in a total of 35 plants grown (**Figures 2A-C**; **Supplementary Table 2**). Genotyping indicated that the non-flowering plants were homozygous for the predicted strongly deleterious mutant alleles *Mtsoc1a-4*, *Mtsoc1b-4*, and *Mtsoc1c-4*, with deletions at multiple guide targets and a large inversion in the case of *Mtsoc1b-4* (**Table 1**). There was 100% co-segregation between the presence of homozygous *Mtsoc1a-4*, *Mtsoc1b-4*, *and Mtsoc1c-4* alleles and the non-flowering phenotype (**Figures 2A-C**; **Supplementary Table 2**).

### Non-flowering triple mutants remain vegetative with a bushy aerial architecture and a short primary axis

**Figure 3:**
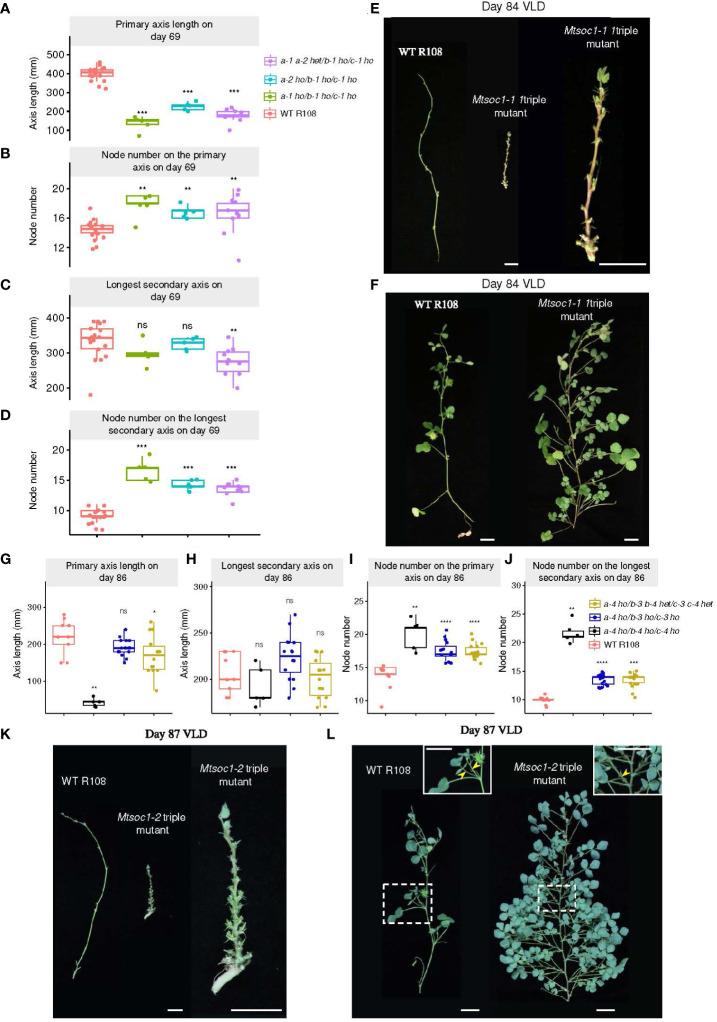
Aerial architecture of plants segregating in triple mutant lines *Mtsoc1-1* and *Mtsoc1-2.* **(A–D)** Aerial architecture of the WT and *Mtsoc1-1* T2 lines (R760 and R761) under VLD, shown as boxplots of primary axis length for each plant **(A)**, Boxplots of number on the primary axis for each plant **(B)**, Boxplots of the longest secondary axis length for each plant **(C)**, Boxplots of the node number on the longest secondary axis for each plant **(D)**. Homozygous (ho) and heterozygous (het) genotypes are shown. **(E)** Left: Photographs of primary axes on day 84 under VLD of the WT and non-flowering *Mtsoc1-1* triple mutant (R760-11), with leaves and branches removed. Right: Close-up view of the *Mtsoc1-1* triple mutant primary axis on day 84 under VLD. **(F)** Photographs of the longest secondary axes on day 84 under VLD of the WT (left) and *Mtsoc1-1* triple mutant (R760-11, right). **(G–J)** Aerial architecture of the WT and *Mtsoc1-2* T2 segregating line (R647) under VLD, shown as a boxplot of the primary axis length for each plant **(G)**, boxplot of the longest secondary axis length for each plant **(H)**, boxplot of the node number on the primary axis for each plant **(I)**, and boxplot of the node number on the longest secondary axis for each plant **(J)**. **(K)** Left: Photographs of primary axes on day 87 under VLD of the WT and non-flowering *Mtsoc1-2* triple mutant (R647-18), with leaves and branches removed. Right: Close-up view of the *Mtsoc1-2* triple mutant primary axis on day 87 under VLD. **(L)** Photographs of the longest secondary axes on day 87 under VLD of the WT (left) and *Mtsoc1-2* triple mutant (R647-18, right). Photographs on the top right are close-ups of the regions in dashed rectangles. Yellow stealth arrows indicate the lateral structures. Scale is 2 cm. Statistical significance was determined using a Wilcoxon test (*P*-value with Bonferroni correction: *P ≤0.05, **P ≤0.01, ***P ≤0.001, ****P ≤0.0001). ns, not significant.

We also scored the aerial architectural phenotype of plants at different ages segregated in the two triple mutant lines (*Mtsoc1-1*, **Figures 3A-F**; **Supplementary Figures 1C–F**; *Mtsoc1-2*, **Figures 3G-L**; **Supplementary Figures 2A–D**). The non-flowering plants had a strikingly short primary axis compared with the WT plants in the VLD (**Figures 1D**, **2C**, **3A, E, G, K**; **Supplementary Figure 1C**, **2A**). These plants were homozygous for *Mtsoc1a-1 Mtsoc1b-1 Mtsoc1c-1* alleles in the *Mtsoc1-1* line or *Mtsoc1a-4 Mtsoc1b-4 Mtsoc1c-4* in the *Mtsoc1-2* line. However, the number of nodes on the primary axis was similar to or greater than that of the WT in these plants (**Figures 3B, E, I, K**; **Supplementary Figures 1E**, **2C**). This indicates that the reduced height of these mutants was not due to slower plant development, as measured by the production of nodes. In contrast, the length of the longest secondary axis was generally similar between the WT and triple mutants (**Figures 3C, F, H, L**; **Supplementary Figure 1D**), except in one experiment (**Supplementary Figure 2B**). However, there was a significantly increase in the node number on the longest secondary axis in the mutant plants at all time points compared to the WT (**Figures 3D, F, J, L**; **Supplementary Figures 1F**, **2D**). This indicated a change in the aerial architecture of the mutant plants, with increased node density relative to the WT on the secondary axes.

The non-flowering *Mtsoc1* triple mutant plants exhibited a bushy phenotype correlated with increased growth of the lateral branch in the leaf axils and increased node density, as noted above, compared with the WT (**Figures 1D**, **2C**, **3F, L**). However, these triple mutants produced only one lateral structure in the leaf axils (**Figure 3L**), which is consistent with the remaining vegetative (Cheng et al., 2021). This contrasts with reproductive WT plants, which produce two structures: a lateral branch and a compound flower in the leaf axils (**Figure 3L**) (Cheng et al., 2021).

### Gene expression analyses indicate that the *Mtsoc1* triple mutants remain vegetative

**Figure 4:**
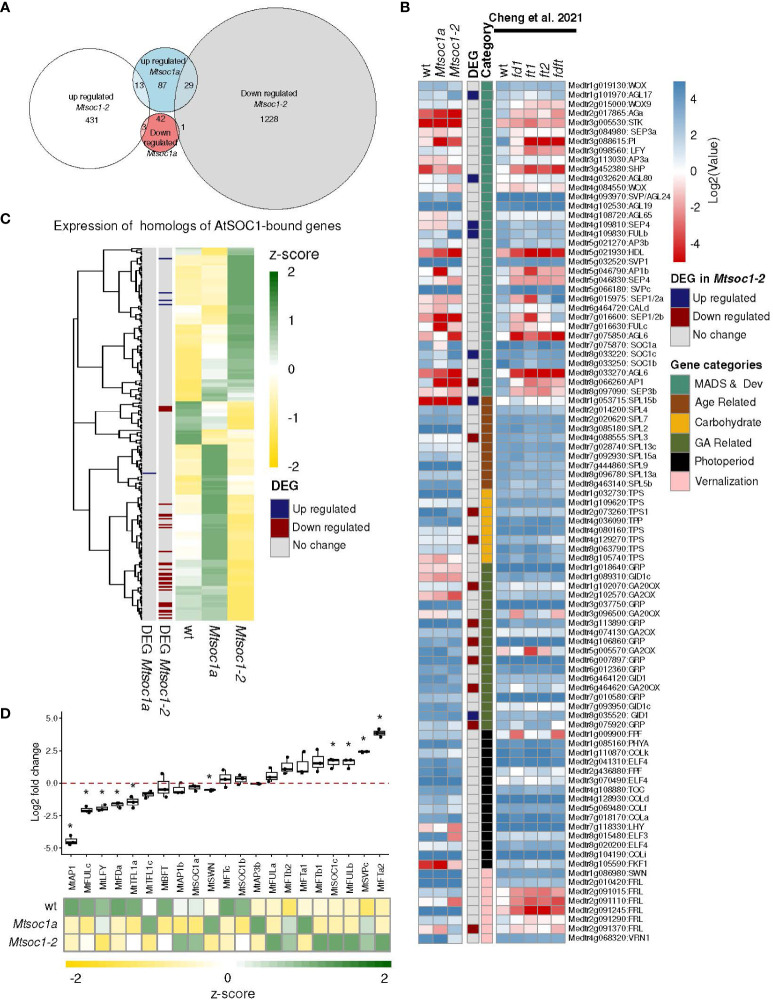
Gene expression in the non-flowering *Mtsoc1-2* triple mutants was consistent with their vegetative phenotypes. **(A)** Euler diagrams showing the number of upregulated and downregulated genes (two-fold change) in the shoot apex of the *Mtsoc1-2* triple mutant (83 days old) and the *Mtsoc1a Tnt1* single mutant (15 days old) compared to WT (15 days old) (*P*_adj_ *<*0.05). **(B)** Expression of Medicago homologs of Arabidopsis genes bound and regulated by AtSOC1 in ChIP-seq from Immink et al. (2012), shown as z-scores extracted from the TPM values. **(C)** Heat map of *Mtsoc1-2* triple mutant (83 days old) and the *Mtsoc1a Tnt1* single mutant (15 days old) and vegetative WT (15 days old) (left, heatmap log2(TPM) is shown), and mutants *Mtfda* (30 days old), *Mtfta1* (30 days old, ft1, 63 days old, ft2), *MtfdaMttfta1* (63 days old), and flowering WT (30 days old) from Cheng et al. (2021) (right part of the heatmap (log2(FKPM) is shown). The 93 genes shown are a list extracted from Cheng et al. (2021) of the selected candidate flowering genes. The DEG in *Mtsoc1-2* genes were upregulated or downregulated with a fold change of 2 and padj ≤0.05. **(D)** Upper panel: RT-qPCR analysis of candidate flowering genes at the apex of the *Mtsoc1-2* triple mutant and WT. Graph showing the relative expression of candidate flowering genes in the apex samples of the *Mtsoc1-2* triple mutant (day 83) and the WT (day 14) under VLD. Relative gene expression was calculated using the formula 2^−ΔCT^, where ΔCT was obtained by normalizing the gene of interest to the reference gene, *MtPP2A*, and presenting the log2 fold change in the mutant relative to WT. the box plot shows three biological replicates. Asterisks indicate significant differences between the mutant and WT strains using the t-test, assuming unequal variance (p ≤0.05). Lower panel: Expression shown as z-scores extracted from the TPM values from RNA-seq.

To investigate the molecular basis of the *Mtsoc1* triple mutant phenotypes, we first compared shoot apex gene expression in non-flowering triple mutants with the *Mtsoc1a* single mutant and WT by RNA-seq. Shoot apical samples were taken from 15-day old *Mtsoc1a* and WT plants and from 83-day-old non-flowering *Mtsoc1-2* triple mutants. We obtained 129 and 447 upregulated genes and 46 and 1,258 downregulated genes in the single and triple mutants, respectively (**Figure 4A**, **Supplementary Table 3**). Consistent with their dramatically different mutant phenotypes (Jaudal et al., 2018), only a few genes (14) were affected in the same way in the two genotypes (13 upregulated and 1 downregulated). We then made a Gene ontology enrichment analysis for the genes up- and down in the two different mutants. We observed that the upregulated genes in the triple mutant were enriched for pathways related to photosynthesis, which is consistent with the vegetative phenotype (Zhang et al., 2021a), and genes that were downregulated were involved in biotic and abiotic stress and hormone-related genes (**Supplementary Table 3**).

Then, to compare the regulation by Arabidopsis SOC1, we asked if the expression of homologs of genes that were bound and regulated by SOC1 was also altered in the Medicago mutants in RNA-seq (**Figure 4B**). We used Blastx to identify the Medicago homologs of a list of AtSOC1-bound and regulated genes identified by Immink et al. (2012) (**Supplementary Table 4**). Strikingly, this indicated that over half of the homologs of the AtSOC1-bound and -regulated genes had altered expression in the non-flowering *Mtsoc1-2* triple mutants (**Figure 4B**). These included two AP2/B3 domain candidate flowering repressors, *MtTEMPRANILLO (MtTEM)1* and *MtTEM2*, which were both significantly downregulated in the triple mutant (**Supplementary Table 4**).

Next, we analyzed the expression of a list of candidate Medicago flowering genes identified by Cheng et al. (2021). In **Figure 4C**, the triple mutant shoot apex (83 days old) was compared with 15-day old *Mtsoc1a* and WT plants, with no visible floral buds (**Supplementary Table 5**). Data from Cheng et al. (2021) on *Mtfta1 Mtfda* double and single mutants and a flowering WT are also shown. Consistent with the vegetative phenotype, MADS genes, including the inflorescence meristem identity gene *MtAP1* and the B class floral organ identity gene *MtPISTILLATA* (*PI*), were downregulated in the *Mtsoc1* triple and *Mtfta1 Mtfda* mutants. Inflorescence identity genes *MtLEAFY* (*LFY*) and *MtFULc* (Cheng et al., 2018) were reduced compared to WT, as were other developmentally important genes required for flowering, such as the *WUSCHEL* homolog *HEADLESS* (*HDL*) (Meng et al., 2018).

Finally, we measured the expression of 21 candidate flowering regulator genes in *Mtsoc1-2* triple mutants (83 days old) and WT shoot apices (14 days old) RT-qPCR (**Figure 4D**, **Supplementary Table 5**). We found that 10 genes were significantly differentially expressed (six upregulated and four downregulated) in the triple mutant compared to the WT in RT-qPCR. These included four key genes that function in the development of the Medicago compound inflorescence; *MtTFL1a* is involved in conferring primary inflorescence (I1) identity, *MtFULc* in secondary inflorescence (I2) identity, *MtAP1* in floral meristem identity, and *MtLFY* is a common primordia determination that gives rise to petals, stamens, and carpels (Benlloch et al., 2015; Cheng et al., 2018). All four genes showed elevated expression levels during the transition to flowering in Medicago (Cheng et al., 2018). Consistent with the vegetative phenotype, *MtAP1*, *MtFULc*, *MtLFY*, and *MtTFL1a* levels were lower in the triple mutant than in the WT. The flowering time and I2 inflorescence meristem identity gene, *MtFDa*, were also downregulated. Four genes, including the likely flowering repressor *MtSVPc* and candidate *FT-like* flowering repressor *MtFTa2* (Jaudal et al., 2022), were upregulated in the triple mutant. RNA-seq analysis of the triple *Mtsoc1* mutant yielded similar results.

## Discussion

### Analysis of *Mtsoc1a Mtsoc1b Mtsoc1c* triple mutants indicates combined critical roles of the three *MtSOC1* genes in Medicago flowering in floral inductive VLD

*SOC1-like* genes are known to play important roles in flowering control in many plants, but *soc1* mutations have not previously been associated with non-flowering phenotypes. For example, in soybeans, mutations in one or both duplicated *GmSOC1* A-class genes, *GmSOC1a* and *GmSOC1b*, led to delayed flowering and increased node number, with *GmSOC1a* having stronger effects, whereas the double mutants showed additive effects on shoot architecture and greater delay in flowering (Kou et al., 2022). Importantly, naturally occurring *GmSOC1a* alleles contribute to optimal latitudinal adaptation in soybean (Kou et al., 2022). Within the *Medicago* genus in legumes, multiple gene duplication events have given rise to three *MtSOC1* paralogs: one Fabaceae A group *MtSOC1a* gene and two B-group genes, *MtSOC1b* and *MtSOC1c*. Here, we describe the generation of *Mtsoc1a Mtsoc1b Mtsoc1c* triple mutant plants by CRISPR-Cas9 gene editing using guides predominantly targeting the exons encoding the K-domain or C-terminal domain. Two independent triple mutant lines, *Mtsoc1-1* and *Mtsoc1-2*, were studied. These lines segregated non-flowering plants in floral inductive VLD. These non-flowering plants were homozygous for the strong mutant alleles of *Mtsoc1a*, *Mtsoc1b*, and *Mtsoc1c*. Phenotyping and gene expression analyses indicated that these non-flowering plants remained vegetative, because they did not transition to flowering. The triple mutant non-flowering phenotype was thus dramatically different from the single *Mtsoc1a Tnt1* mutant, which had a moderate delay to flowering of ~10 days compared with the WT in VLD (Jaudal et al., 2018). These results imply a combined critical role of multiple *MtSOC1* genes in the transition to flowering in Medicago.

### *MtSOC1* genes and primary axis elongation

The non-flowering triple mutants had shorter primary axes than the WT (**Figures 3E, K**), consistent with previous studies implicating *MtSOC1a* in primary axis elongation. The *Mtsoc1a Tnt1* single mutant had a shorter primary axis than the WT, while overexpression of *MtSOC1a* caused increased primary axis elongation compared with the WT (Jaudal et al., 2018). Interestingly, in the *Mtsoc1-1* line, the primary axis was also shorter in plants homozygous for the strong *Mtsoc1b-1* and *Mtsoc1c-1* alleles but heterozygous or homozygous for the predicted weaker *Mtsoc1a-2* allele (**Figure 3A**; **Supplementary Figures 1C**, **2A**; **Table 1**). *Mtsoc1a-2* is predicted to affect the C-terminal domain, which is highly variable in sequence between different MADs transcription factors (Lai et al., 2021) and has a weak effect on flowering in the triple mutant (**Figure 1C**, **Supplementary Figures 1A, B**). Therefore, the *Mtsoc1b-1* and *Mtsoc1c-1* alleles likely contribute strongly to the short primary axis phenotype of the triple mutant, which correlates with the strong expression of *MtSOC1b* and *MtSOC1c* in primary axis stems (Fudge et al., 2018).

### The non-flowering *Mtsoc1* triple mutants appear to remain vegetative

The non-flowering triple mutants were also bushy in appearance because of the increased node density observed on the secondary axes, and increased growth of the lateral branch in their leaf axils (**Figure 3**). They only produce one lateral structure, a lateral branch in their leaf axils (**Figure 3L**) indicating that they remain vegetative in the VLD (Cheng et al., 2021). The non-flowering triple mutant phenotype thus appears like the *Mtfta1 Mtfda* double mutant that failed to transition to flowering (Cheng et al., 2021). Similar to the triple mutants, *Mtfta1Mtfda* double mutants remained vegetative with a bushy phenotype, producing only one lateral branch in the leaf axils. In contrast, the non-flowering *Mtfulc* mutant does transition to flowering but cannot produce flowers. Instead, it produces two lateral branches in the leaf axils due to the aberrant elevation of *MtTFL1a* in the secondary inflorescence meristem (Cheng et al., 2018).

Gene expression analysis also supported the idea that the triple mutants remained vegetative. Four key genes that promote the development of Medicago compound inflorescence, *MtAP1*, *MtTFL1a*, *MtLFY*, and *MtFULc*, were downregulated in the apex of the triple mutant, consistent with the remaining vegetative cells (**Figure 4D**) (Cheng et al., 2018, 2021). *MtFDa* was also downregulated at the apex of the triple mutant. The candidate floral repressors *MtFTa2* and *MtSVPc* were upregulated in the triple mutant (**Figure 4D**), consistent with their elevation in the late-flowering *Mting2* mutant (Jaudal et al., 2022), indicating that they are likely to play a repressive role in Medicago flowering. *MtFULb* and *MtSOC1c* levels were elevated in the triple mutant, suggesting their misregulation in the shoot apex. Other MADS-box genes that are likely to promote floral meristem and floral organ identity and/or development were also downregulated in the triple mutant shoot apex, consistent with the non-flowering phenotype (**Supplementary Table 3**). These include genes homologous to *SEP*, *AGL6*, *B* function genes *PI* and *AP3*, *AG-like* C function genes, and a predicted D function gene, *MtSEEDSTICK-like* (*STK*).

### Perspectives

While the three *MtSOC1* genes are likely to function predominantly downstream of *MtFTa1* and *MtFDa* in promoting the vegetative-to-reproductive transition (Fudge et al., 2018; Jaudal et al., 2018; Cheng et al., 2021; Zhang et al., 2021b), we found that *MtFDa* gene expression was also reduced in the *Mtsoc1a* single mutant and the triple mutant. This may indicate negative feedback regulation of MtSOC1s on *MtFDa*, consistent with the multiple interactions observed between *SOC1* and flowering regulators in Arabidopsis (Immink et al., 2012; Tao et al., 2012). It is also possible that *MtSOC1* genes function in both floral induction and I1 and/or I2 inflorescence development, as has been reported for *MtFDa* (Cheng et al., 2021; Zhang et al., 2021b). One mechanism by which MtFDa regulates inflorescence development is via stimulation of *MtFULc* expression in I2, which in turn represses *MtTFL1a* in I2, enabling floral meristem formation via activation of *MtPIM* (Cheng et al., 2021; Zhang et al., 2021b). The *MtSOC1a* transcript was detected in I1 and I2 primordia via *in situ* hybridization in the WT (Jaudal et al., 2018). In addition, a sharp elevation of *MtSOC1b* transcripts in the shoot apex was detected at flowering by RT-qPCR, later than the increase in expression observed for *MtSOC1a* and *MtSOC1c* (Jaudal et al., 2018). This indicates potential additional roles for *MtSOC1s* downstream of floral induction that remain to be uncovered. Taken together, our phenotyping and global transcriptomic analyses implicated multiple *MtSOC1* genes in overlapping and complementary functions, which are essential for the vegetative-to-floral transition in VLD and the development of normal aerial architecture, unlike Arabidopsis *SOC1* (Lee and Lee, 2010) or *MtSOC1a* alone (Jaudal et al., 2018). Ultimately, the non-flowering phenotype may be used to improved forage and biofuel productivity in temperate legumes (Jung and Muller, 2009; Tadege et al., 2015; Wolabu et al., 2023).

## Data availability statement

The datasets presented in this study can be found in online repositories. The names of the repository/repositories and accession number(s) can be found in the article/**Supplementary Material**.

## Author contributions

AP: Conceptualization, Data curation, Formal analysis, Visualization, Writing – original draft, Writing – review & editing. MZ: Investigation, Writing – review & editing. YP: Data curation, Investigation, Project administration, Writing – original draft, Writing – review & editing. FT: Data curation, Investigation, Writing – review & editing. MJ: Conceptualization, Methodology, Writing – review & editing. LZ: Investigation, Methodology, Writing – review & editing. JW: Funding acquisition, Writing – review & editing. JP: Conceptualization, Funding acquisition, Project administration, Resources, Supervision, Writing – original draft, Writing – review & editing.

## Supplementary material

Supplementary Table 1 List of primers and guides.

Supplementary Table 2 Lists of plant genotypes and flowering time.

Supplementary Table 3 RNA-seq analysis for *Mtsoc1a* and *Mtsoc1-2* triple mutant and Gene Ontology analysis.

Supplementary Table 4 Expression of Medicago homologs of AtSOC1-bound and regulated genes listed in Immink et al. (2012).

Supplementary Table 5 Expression of candidate flowering genes listed in Cheng et al. (2021) and genes used in RT-qPCR.
