# Supplementary figures and images for: Gene-edited Mtsoc1 triple mutant Medicago plants do not flower
Source: Front Plant Sci. 2024 Feb 26;15:1357924. doi: 10.3389/fpls.2024.1357924 (PMC10926907; doi:10.3389/fpls.2024.1357924)

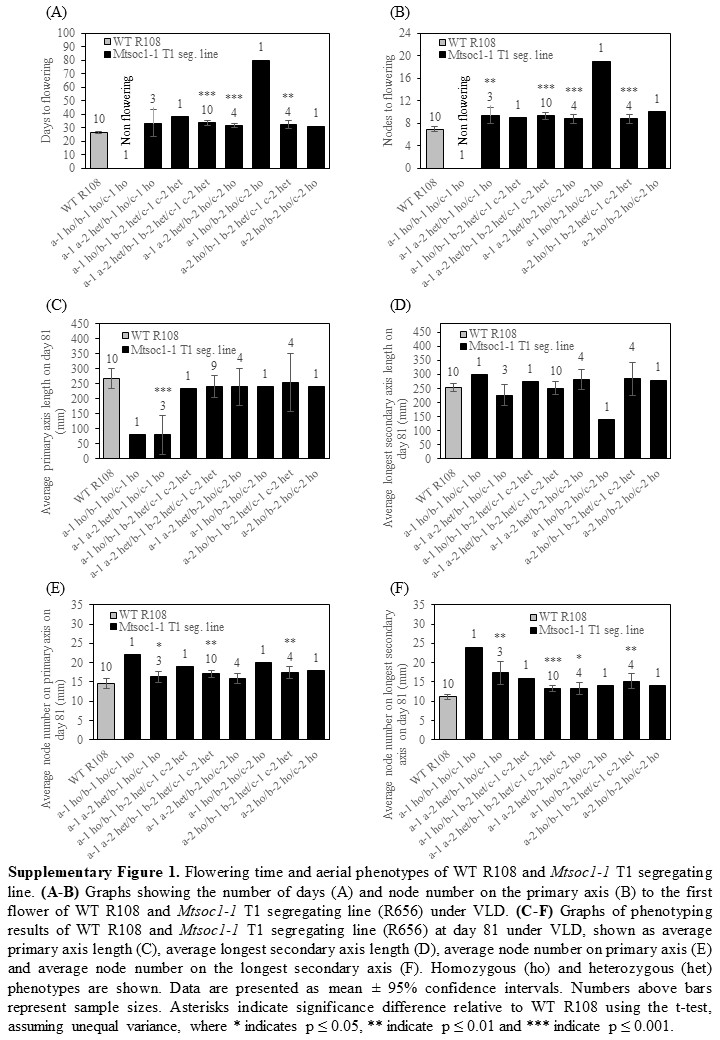

Supplement: Supplementary file 1 [file Image_1.jpeg]

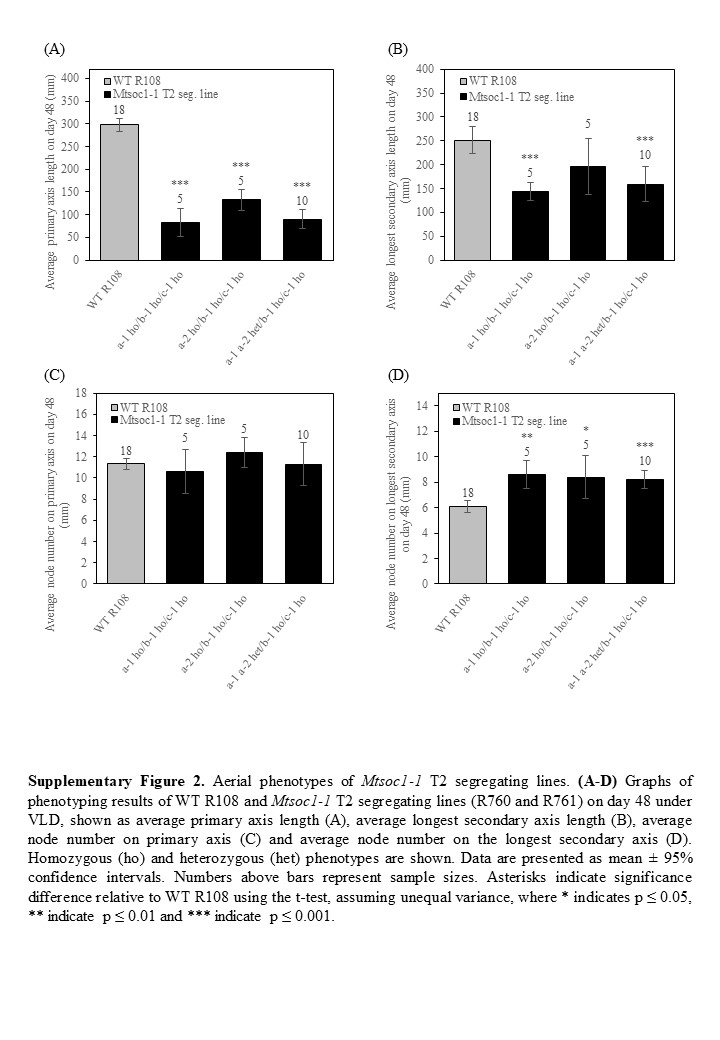

Supplement: Supplementary file 2 [file Image_2.jpeg]
